# A novel phosphorylation by AMP-activated kinase regulates RUNX2 from ubiquitination in osteogenesis over adipogenesis

**DOI:** 10.1038/s41419-018-0791-7

**Published:** 2018-07-09

**Authors:** Suresh Chava, S. Chennakesavulu, B. Meher Gayatri, Aramati B. M. Reddy

**Affiliations:** Department of Animal Biology, School of Life Sciences, University of Hyderabad, Hyderabad, 500046 India

## Abstract

Mesenchymal stem cells (MSCs) function as progenitors to a variety of cell types. The reported association between osteogenic and adipogenic commitment during differentiation is due to the regulation of key transcription factors in the signaling pathways. However, the process of adipogenesis at the expense of osteogenic phenotype during metabolic stress is still unclear. In this study, we showed for the first time that RUNX2 is a novel substrate of AMP-activated kinase (AMPK), which directly phosphorylates at serine 118 residue in the DNA-binding domain of RUNX2. Our results in in vitro MSC lineage differentiation models confirmed that active AMPK and RUNX2-S118 phosphorylation are preferentially associated with osteogenic commitment, whereas the lack of this phosphorylation leads to adipogenesis. This interplay is regulated by the ubiquitination of non-phosphorylated RUNX2-S118, which is evident in the dominant mutant RUNX2-S118D. Pharmacological activation of AMPK by metformin significantly abrogated the loss of RUNX2-S118 phosphorylation and protected from tunicamycin-induced endoplasmic reticulum stress, high glucose-induced in vitro adipogenesis and streptozotocin-induced in vivo bone adiposity and bone phenotype. In conclusion, results from this study demonstrated that RUNX2 is a direct target of AMPK which simplified the outlook towards several complex mechanisms that are currently established concerning cellular metabolism and pathogenesis.

## Introduction

Bone marrow functions as a continuous source for hematopoiesis^1^. Critical regulation of complex signaling mechanisms is important in maintaining normal homeostasis of multi-lineage commitment^1,2^. Loss of normal structure of bone and mineral density is usually associated with imbalance in homeostasis of lineage commitment, which is often seen in metabolic disorders such as diabetes^3–6^. Bone marrow-derived mesenchymal stem cells (MSCs) are non-hematopoietic that serve as progenitors for adipogenic, osteogenic, chondrogenic and myogenic differentiation^2^. Hyperglycemia or stress-induced signaling have shown to favor adipocytes over osteoblast lineage commitment, thus leading to bone adipogenesis or osteoporosis phenotype^7^. Several studies reported that the reciprocal relationship between adipogenesis and osteogenesis from MSCs is determined by a combination of regulatory signaling pathways induced by bone marrow niche; however, there are some missing links that exit within this complex stimuli. It has been appropriately demonstrated that lineage-specific transcriptional regulators, RUNX2, DLX5 and Osterix, favor osteoblasts^8,9^, whereas the CEBP (CCAAT/enhancer binding protein) family (α, β, δ) and PPARγ2 (peroxisome proliferator-activated receptor-γ2) favor adipocytes^10,11^. However, the commitment of adipogenesis over osteogenesis or vice versa and the transdifferentiation process of the committed cell type, particularly under pathological conditions, is still not clear. Understanding the detailed molecular mechanisms, which would explain how the balance is maintained, may provide insights into the role of these transcription factors in normal MSC differentiation and also transdifferentiation process during metabolic stress leading to disease phenotype. Thus, RUNX2 and PPARγ play a central role in understanding the molecular mechanism that control the regulation of osteogenesis vs adipogenesis.

It has been demonstrated that AMP-activated kinase (AMPK) is a cellular energy sensor and acts to maintain energy homeostasis by diversifying the signaling mechanisms related to catabolic vs anabolic processes for cell survival; therefore AMPK participates in highly conserved signaling pathways^12^. Recent studies indicate that AMPK activation stimulates bone formation by inhibiting adipogenesis^13–17^ and loss of AMPK signaling leads to altered downstream signaling and induces pro-inflammatory cytokines that potentially causes insulin resistance. Recent studies demonstrating the phosphorylation of AMPK stimulates bone formation signifies its role in defining the mesenchymal differentiation^18^. However, the molecular connection between AMPK activation and osteocyte differentiation is still unclear.

In this study we have demonstrated that RUNX2 is a novel substrate for AMPK, and activation of AMPK directly phosphorylates RUNX2. AMPK activity in various differentiation and transdifferentiation models and diabetes-induced bone adipogenesis studies exhibited that both AMPK and RUNX2 are synergistically regulated during adipogenesis and osteogenesis, further, this study shows that RUNX2-S118 phosphorylation is critical. Therefore, this study demonstrates the important molecular mechanisms that have not been explored so far in the AMPK–RUNX2 circuit, which also exhibit a significant impact on the biology of metabolic and malignant conditions, where AMPK and RUNX2 are known to be important targets.

## Materials and methods

### In silico analysis

AMPK-dependent phosphorylation prediction was performed using PhosphoMotif Finder, and its phosphorylation efficiency was measured by NetPhos 3.1 tool. RUNX2 protein sequence alignments were done by ClustalW2. All the sequences were taken from the NCBI (National Center for Biotechnology Information) human database and numbering also corresponds to human RUNX2 (NM_001024630.3).

### Cell culture and reagents

The multipotent murine MSCs (C3H10T1/2), murine 3T3-L1 pre-adipocytes, murine skeletal muscle cells (C2C12) and human embryonic kidney cells (HEK) 293T cells were procured from ATCC (Manassas, VA, USA). All cells were maintained as per ATCC protocol. Tunicamycin, MG132, rotenone, thapsigargin, AMPK activators (metformin, AICAR) and inhibitors (compound C) were procured from Sigma, USA.

### Bone marrow-derived mesenchymal stem cell (BM-MSC) isolation

BALB/c male mice (6-8 weeks) were killed by cervical dislocation and the whole mice were wiped with 70% ethanol for 5 min. BM-MSCs were isolated as described earlier^19^. In brief, the fore limbs and hind limbs were dissected at the ankle and carpel joints before removing the soft tissues associated with tibiae and femur. The ends of marrow cavity was excised followed by flushing with 5 ml of Iscove's modified Eagle's medium (IMEM) using 23 G needle. For culturing BM-MSCs, the collected cells were rinsed with IMEM containing 1× antibiotics before plating in 100 mm tissue culture dish and incubating in 37 °C and in 5% CO_2_ for 5 days. The medium was replaced every 72 h. The cultures were passaged (0.25% trypsin for 2 min) after reaching 70–80% confluency. After the third passage the cells characterized by fluorescence-activated cell-sorting analysis by staining with CD44, CD90 (positive MSC markers) and CD45 (negative MSC marker) (Abcam, Cambridge, MA) (Figure S1). Well-characterized BM-MSCs were used for all experiments, whereas for RNA and protein expression analysis on bone marrow flush cells in normal, diabetic and metformin-treated mice, the collected cells were directly subjected for Trizol or RIPA lysis respectively.

### Construction of plasmids

WT RUNX2 cDNA (NM_001024630.3) was commercially obtained from Genecopoeia, USA (H5214). WT, site-specific S118A, R115A and deletion mutants; 1–140, 1–140 S118A, 141–521 of RUNX2 were generated and sub-cloned into glutathione-*S*-transferase (GST)-tagged vector pGEX-4T1 (GE Health Care) for bacterial expression. Whereas, RUNX2-WT, site-directed mutants of S118A, S118D and R115A were cloned into pDsRed1-N1 (Clonetech, USA) containing red fluorescent protein for mammalian expression. List of primers used for real-time PCR (RT-PCR) analysis and site-directed mutagenesis are given in Supplementary Table S1.

### Bacterial protein expression and purification

GST-fused RUNX2-WT, RUNX2-S118A, RUNX2–R115A, RUNX2 (1–140) and RUNX2 (1–140)-S118A proteins were expressed in *Escherichia coli* (BL21) strain along with vector control (pGEX-4T1) by inducing with 1 mM IPTG at 16 °C overnight. Supernatants of sonicated cell-free extracts were allowed to bind onto an equilibrated column packed with GST beads, washed with cold column wash buffer and eluted with 25 mM reduced glutathione. Eluted protein fractions were analyzed by 10% sodium dodecyl sulfate–polyacrylamide gel electrophoresis (SDS-PAGE).

### In vitro AMP-activated serine threonine protein kinase assay

The reaction mixture containing protein (5 μg of GST-RUNX2-WT, RUNX2-S118A, RUNX2–R115A, RUNX2 (1–140), RUNX2 (1–140) S118A or GST protein), kinase assay buffer (20 mM HEPES, pH 7.4, 100 μM cold ATP and 5 μCi [γ-^32^P] ATP for autoradiograms, 1 mM dithiothreitol, 10 mM magnesium acetate) along with 100 ng of AMPK purified enzyme (Cat #14-840 – EMD Millipore) was incubated for 30 min  at 30^0^C. The assay contents were subjected for SDS-PAGE and were exposed to autoradiography and non-radiography reactions for western blot analysis using AMPK substrate-specific antibody (Cell Signaling, USA).

### Diabetic animal models

BALB/c male, 6–7-week-old mice were procured from National Animal Resource Facility for Biomedical Research (NARFBR), NIN (Hyderabad, India) and housed at Animal Resource Facility at University of Hyderabad, India, for acclimatization before beginning of the experiments. All the animal procedures were performed adhering to the norms of Institutional Animal Ethics Committee regulated by the Committee for the Purpose of Control and Supervision of Experiments on Animals (CPCSEA) of India.

Diabetes was induced by daily intraperitoneal injections of streptozotocin (40 mg/kg body weight), in citrate buffer pH 4.5 for 5 days and controls were injected with citrate buffer alone. After a week of the last dose, the blood glucose (non-fasting) level was measured with the glucometer (Accu-Check, Roche Corp., Indianapolis, IN) and the mice exhibiting more than 300 mg/dl blood glucose were classified as the diabetic group. Mice were killed at 10 weeks after confirmation of diabetes. Metformin control and diabetes treated with metformin group mice received 60 mg/kg body weight of metformin daily intraperitoneally for 10 weeks. All the mice were killed and subjected to analysis.

### Tissue and bone histology and histomorphometry

Proximal tibiae were collected from all the mice after appropriate treatments and subjected to histology as described earlier. In brief, the fixed samples were decalcified in 5% nitric acid and were embedded in paraffin, and slides were prepared. Hematoxylin and eosin (H&E) staining was performed to observe the histology. Visible adipocytes, greater than ~30 µm, were observed.

### Micro-computed tomography (micro-CT) analysis

Femurs and tibiae from normal, diabetic and diabetic treated with metformin were scanned (*n* = 3/group) with a SkyScan 1176 system (Bruker, Billerica, MA) at a pixel size of 12.34 µm, with a source voltage and current of 50 kV, 500 µA, correspondingly. A 0.5 mm Al Filter was used to reduce the beam hardening from the polychromatic nature of the covered X-ray source. Subsequent scanning, three-dimensional microstructural images of the sagittal and axial planes of the tibia metaphysis and diaphysis were generated and recreated using SkyScan CTvox, CTAn softwares. Volumes of interest were distinct and structural indices calculated using SkyScan CT Analyzer software. The regions of interest for trabecular micro-architectural parameters were used to quantify percent of trabecular bone volume (BV/TV), mean trabecular number (Tb. N), mean trabecular thickness (Tb.Th) and bone surface density (BS/TV).

### Differentiation/transdifferentiation

For adipogenic differentiation from either mesenchymal stem cells (BM-MSCs or C3H10T1/2) or pre-adipocytes (3T3-L1), 85–90% confluent cells were induced with regular growth medium supplemented with 10% fetal bovine serum (FBS), 0.5 mM IBMX, 20 nM insulin and 0.1 µM dexamethasone (Sigma) for 48 h and the medium was replaced with fresh medium containing 10% FBS and 20 nM insulin for an additional 8 days for C3H10T1/2/ 3T3-L1 and 16 days for BM-MSCs by replacing with fresh medium every alternate day.

For transdifferentiation, the osteoblast cells (U2OS) were induced by culturing cells in the medium for 14 days. FBS was substituted with charcoal stripped FBS (10%), and Rosiglitazone (1 µM) (Sigma) was added starting post confluent day 4 and maintained for the next 14 days. In both differentiation and transdifferentiation, the adipogenesis was confirmed by Oil Red O staining. Cell lysates from different sets were collected at different time intervals and subjected to immunoprecipitation/western blotting.

### Oil Red O staining

Oil Red O staining solution was prepared by diluting with distilled water (6:4) from stock solution of 0.5% Oil Red O (Sigma–Aldrich) dissolved in isopropanol overnight. After appropriate incubations, phosphate-buffered saline (PBS)-washed cells were fixed with 4% formaldehyde for 1 h at room temperature. The excess stain was removed by PBS and 60% isopropanol wash before eluting with 100% isopropanol for quantification at 500 nm.

### Immunoprecipitation and western blotting

Immunoprecipitation was done by following the procedure described in earlier studies;^20^ In brief, equal amounts (750 μg) of cell lysates obtained after the indicated treatments were incubated with α-RUNX2 or α-p-AMPK (cell Signaling) at 4 °C overnight. Protein A-Sepharose resin beads (2.5 mg/sample) (GE Healthcare) were used to pull the immunoprecipitated protein complexes and were subjected to western blotting. Where immunoprecipitation was not done, cell lysates were directly subjected to western blotting and probed with respective specific antibodies α-RUNX2, α-β-Actin, α-p-AMPK, α-Bip1, α-p-GSK3β, α-p-JNK, α-p-AMPK substrate motif-specific antibodies and respective horseradish peroxidase enzyme-linked secondary antibodies obtained from Cell Signaling Technology, USA. The α-PPARγ was obtained from Santa Cruz Biotechnology (Santa Cruz, CA, USA). The signal was detected by the Amersham ECL prime Western blotting detection reagent, and images were documented by Bio-Rad chemidoc MP system.

### Confocal microscopy analysis

Confluent (80%) cells, cultured on coverslips, were treated with indicated agents and time points and were washed with PBS before fixing in 4% formalin for 10 min at room temperature. The slides were stained for primary antibodies α-p-AMPK, α-RUNX2 (CST #8486) and α-ubiquitin antibody (SCBT, #SC-8017) and respective fluorescence secondary antibodies (Alexa Flour 488 and Alexa Flour 546) by following standard procedures. Slides were counterstained with 4′,6-diamidino-2-phenylindole (DAPI) for nuclei and the images were captured using a laser scanning confocal microscope (LSM 780, Carl Zeiss).

### Electrophoretic mobility shift assay (EMSA)

HEK 293T cells were transiently transfected with RUNX2-WT, RUNX2-S118A and RUNX2-S118D clones in pDsRed-N1 vector using lipofectamine 2000 (Invitrogen, USA). Cells of appropriate groups were treated with metformin (5 mM) and compound C (20 µM) for 6 h. The nuclear extracts were separated using standard procedure as described earlier^21^. The RUNX2 binding consensus sequence at -140 (5′-CGAGTATTGTGGTTAATACG-3′) on Osteocalcin promoter was labeled with [γ-^32^P] and used as EMSA probe. The nuclear extract (5 µg) was incubated with 10 pmol of probe at 37 °C for 30 min in 1× binding buffer (200 mM HEPES (pH 7.4), 40 mM dithiothreitol, 4 mM EDTA (pH-8.0) and 50% glycerol) and these nuclear protein complexes were loaded on 7.5% native acrylamide gel and the dried gels were exposed for autoradiogram

### Statistical analysis

The data values are expressed as mean ± SEM. Statistical analysis was performed using Student's *t*-test. The difference between the mean values was analyzed using Student's *t*-tests. The *P* value of less than 0.1 was considered to be statistically significant. For all cell culture studies, a minimum of three independent experiments were carried out. For all mice-related work, each mouse was considered as an experimental component.

## Results

### RUNX2 is a novel substrate of AMPK

**Fig. 1:**
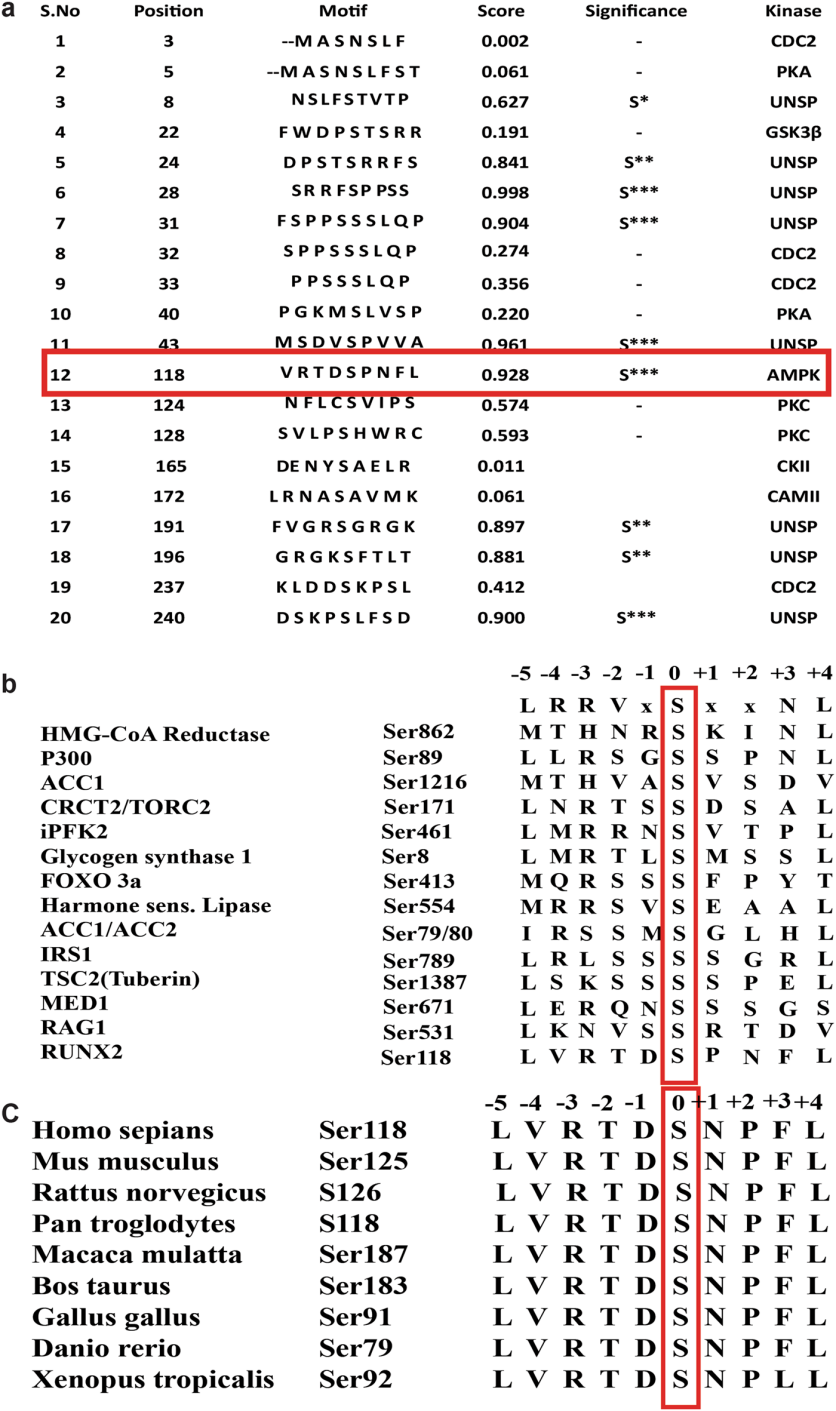
In silico prediction of AMPK phosphorylation site in RUNX2 protein. **a** List of corresponding putative motif-specific kinases along with AMPK predicted by phospho motif finder in the N-terminal region (DNA-binding domain) of RUNX2. **b** The predicted corresponding putative AMPK substrate motif of RUNX2 significantly aligned with all the important residues of known and validated AMPK substrate motifs. **c** The ClustalW2 sequence alignment showing cross-species conservation with predicted optimal AMPK substrate motif of RUNX2

RUNX2 is a transcription factor involved in skeletal development and is also essential in self-renewal of MSCs^22^. Recent studies on 3T3-L1 demonstrated that knockdown of RUNX2 significantly altered gene networks associated with insulin signaling and energy homeostasis^23^. Since AMPK is a master regulator of energy homeostasis, to check the functional correlation between RUNX2 and AMPK, we first performed in silico analysis using PhosphoMotif Finder tool^24^ to predict putative kinase motif on RUNX2^25^. The algorithm of this tool predicted an AMPK-binding motif at 113 to 122 amino acids with target of serine 118 having the highest motif consensus score (Fig. 1a). The motif consists of highly preferred basic amino acids at –3 positions, which act as phosphoacceptor site. The other consensus hydrophobic residues including leucine and methionine at –5 and +4 positions, respectively, are known to be significant for strong substrate selectivity as demonstrated earlier by site-directed mutagenesis studies and molecular modeling studies^26^. This prediction was further supported by NetPhos 3.1 tool analysis, which exhibited a highly preferable score. However, the other predicted kinases at the motif targeting serine 118 by PhosphoMotif finder did not show any homology consensus/conservation (data not shown), whereas predicted AMPK motif on RUNX2 is highly conserved with other known substrates of AMPK and cross-species conservation of RUNX2 protein (Fig. 1b, c).

**Fig. 2:**
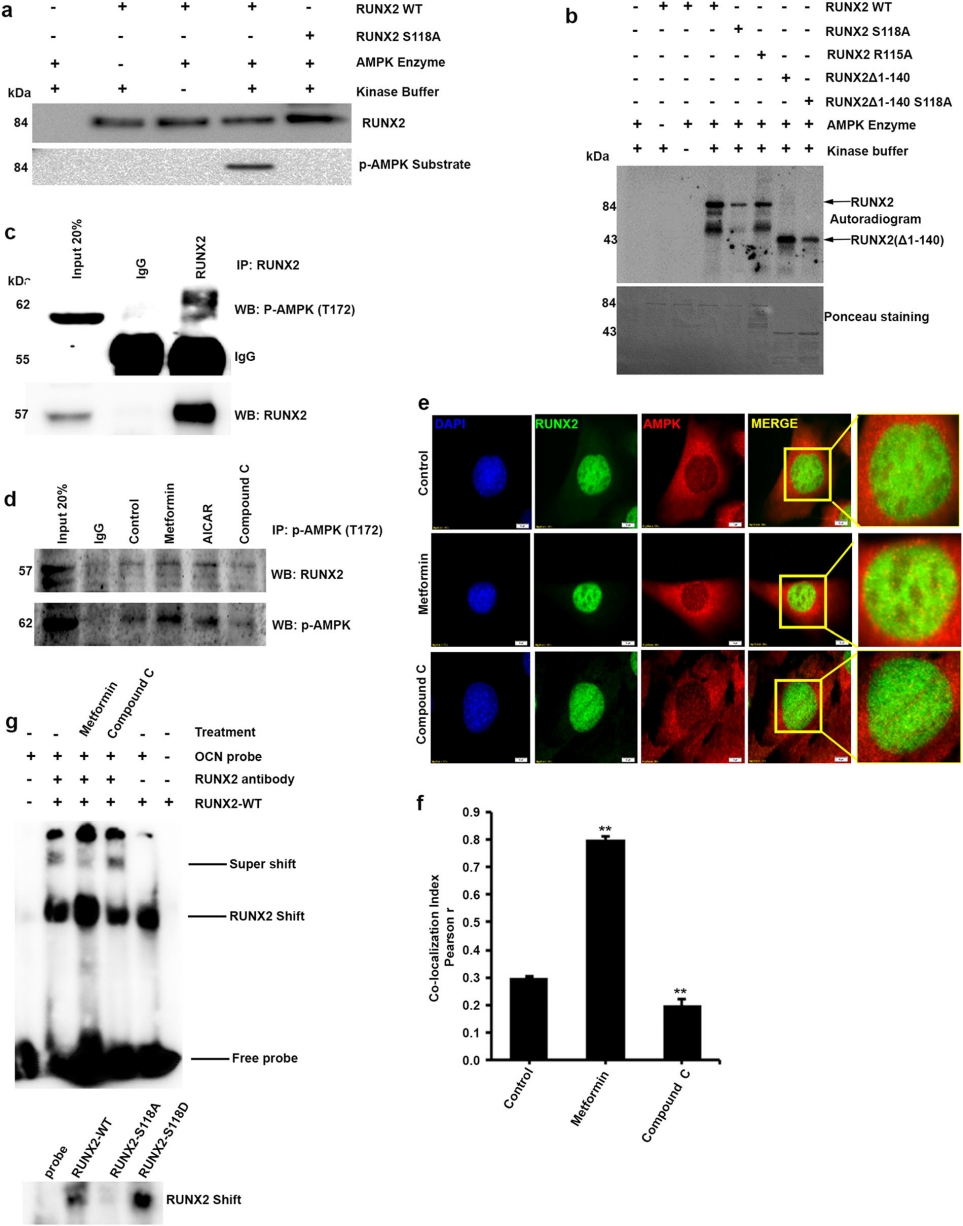
In vitro phosphorylation of RUNX2 is AMPK specific. **a** p-AMPK substrate motif-specific antibody showing phosphorylation of GST-RUNX2-WT but not in lanes of serine-specific mutant of RUNX2-S118A, and GST control and **b** confirmed by [γ-^32^P] autoradiogram. Phosphorylation bands were detected in N-terminal protein (1–140) lane only. Lane RUNX2–R115A shows decreased intensity bands and explains the significance of highly conserved charged amino acids in efficient binding of AMPK. **c** Immunoprecipitation analysis showing the physical interaction of endogenous RUNX2 with AMPK. **d** Increased immunoprecipitation was seen by active AMPK with RUNX2 in either metformin or AICAR-treated C2C12 cells compared with compound C treatments. **e** Colocalization studies by immunofluorescence staining confirms the physical interaction, RUNX2 (Alexa Fluor 488) AMPK (Alexa Fluor 546) and Nuclei (DAPI) and **f** the quantification was performed using Image-J software on three independent experiments and fields. **g** Electrophoretic mobility shift assay was performed using nuclear lysates from RUNX2 full length overexpressed followed by metformin (5 mM) and compound C (20 µM) treatment in HEK 293T cells. The subsequent nuclear extracts overexpressed (RUNX2-WT, S118A and S118D) were incubated with osteocalcin promoter-specific probe. Mean  ± S.E.M.; n = 3,**p < 0.01 versus control

To validate our in silico observations, we performed AMP-activated kinase assay in a cell-free system using partially purified site-specific motif mutants (S118A and R115A) of GST-RUNX2 along with wild-type protein as described in the materials and methods section. Western blot analysis of in vitro kinase assayed proteins using p-AMPK substrate motif-specific antibody shows that AMPK efficiently phosphorylated GST-RUNX2-WT but no phosphorylation was observed in serine 118 mutant of RUNX2 (GST-RUNX2-S118A) (Fig. 2a), which was further confirmed by autoradiography. The results showed phosphorylation in both RUNX2-WT and RUNX2-Δ1-140, but reduced phosphorylation in RUNX2-S118A, RUNX2-Δ1-140-S118A and the R115A mutant at –3 position of conserved residue in AMPK motif site^26^, emphasizing that the motif is highly specific (Fig. 2b). S118 phosphorylation was also confirmed by mass spectrometry analysis on in vitro kinase assay sample (data not shown). Follow-up immunoprecipitation experiments in myoblasts cells (C2C12) showed that the pharmacological activation of AMPK using metformin or AICAR (5-aminoimidazole-4-carboxamide ribonucleotide) resulted in co-precipitation of p-AMPK with RUNX2, and this was not observed in AMPK inhibitor (compound C)-treated cells (Fig. 2c, d). We characterized these observations further by immunofluorescence studies where colocalization of AMPK and RUNX2 were identified in metformin-treated cells (Fig. 2e, f). Overall, these findings confirm our in silico prediction of RUNX2 as a physiological substrate of AMPK. Since the identified AMPK-binding motif (S118) was present in DNA-binding domain of RUNX2, we have assayed to check the effect of S118 phosphorylation on DNA-binding activity of RUNX2 by EMSA. The results demonstrated that metformin-induced and phosphomimetic S118D mutant-overexpressed lysates showed an enhanced binding activity of RUNX2 to its consensus DNA sequence (Fig. 2g).

### Adipogenesis is associated with AMPK-mediated loss of RUNX2-S118 phosphorylation in transdifferentiation models

**Fig. 3:**
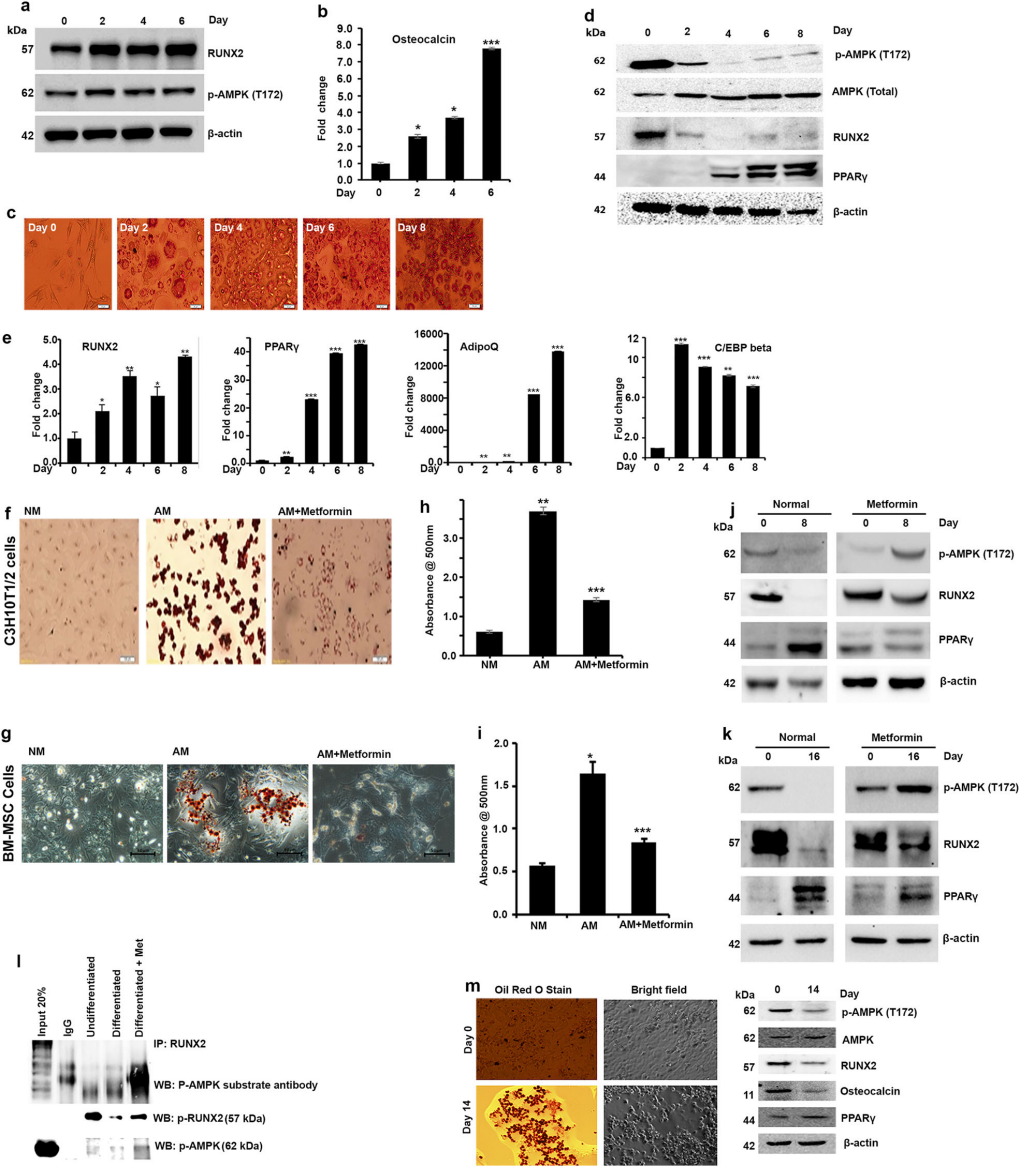
Synergistic association of phospho-AMPK and RUNX2 protein levels in differentiation and transdifferentiation models. **a** Western blot analysis showing differentiation of myoblasts (C2C12) to osteogenesis was associated with increased RUNX2 and p-AMPK (T172) and **b** RT-PCR analysis confirms expression of RUNX2 downstream target, osteocalcin. **c** Adipogenesis was confirmed by staining for oil droplets using Oil Red O stain (3T3-L1) at different time points during differentiation. **d** Western blots showing decreased phosphorylated AMPK, which synergistically correlated with low levels of RUNX2 protein. Time-dependent increased expression of PPARγ was seen with adipogenesis. RT-PCR analysis showing **e** increased RNA levels of RUNX2 along with AdipoQ, PPARγ and C/EBPβ. **f**, **g** Activation of AMPK by metformin for 48 h before treating with adipogenic differentiation medium abrogated the effects of adipogenic inducers when compared with control (NM), and adipogenic medium (AM) alone in MSCs was evident from the reduced oil droplets (dark brown). **h**, **i** Representative picture of quantitative measurements of eluted Oil Red O stain precipitates (*n* = 3). **j**, **k** Western blot analysis for p-AMPK, RUNX2 and PPARγ expression. **l** Immunoprecipitation analysis of differentiated MSCs (C3H10t1/2) to adipogenesis with and without metformin showing the reduced levels of RUNX2, p-AMPK and RUNX2-S118 phosphorylation. **m** Oil Red O staining and western blot confirms the transdifferentiation of osteocytes (U2OS) to adipocytes after induction with adipogenic medium containing charcoal stripped FBS (10%) and rosiglitazone (1 µM) for 14 days. Mean ± S.E.M.; *n* = 3, *p < 0.1 versus control, **p < 0.05 versus control; ***p < 0.001 versus control

Recent studies have demonstrated that AMPK may have a role in MSC differentiation; however, the mechanistic association and downstream signaling events remain unclear^3^. As our in silico and in vitro analyses confirm that RUNX2 is a substrate for AMPK, we have made an attempt to understand the significance and relationship between AMPK activation and RUNX2-S118 phosphorylation during the differentiation process. To demonstrate the same, we utilized various well-characterized differentiation models to study downstream signaling mediators in the osteogenesis vs adipogenesis process. Our data show that phosphorylated (active) form of AMPK (T172), expression of RUNX2 and increased mRNA level of Osteocalcin is associated throughout the differentiation of osteoblasts from myoblasts (C2C12) upon induction with BMP2 (Fig. 3a, b). We then examined the physiological functions of AMPK-induced RUNX2 phosphorylation and its downstream effects in pre-adipocyte-to-adipocyte differentiation using 3T3-L1 as described in the Materials and methods section. By day 8 from the induction of differentiation, RUNX2 protein level and AMPK activity were decreased whereas that of other adipogenic markers such as PPARγ, AdipoQ and C/EBPβ were increased as shown in Oil Red O staining, western blot and real-time PCR analysis (Fig. 3c–e). These results demonstrated that adipogenesis is associated with loss of RUNX2 protein level and active AMPK.

As it is evident from the above results, the loss of AMPK activity and RUNX2 expression are associated with adipocyte differentiation in both MSC and 3T3-L1 pre-adipocyte models, and we then examined the effects of pharmacological activation of AMPK in adipogenesis in MSC differentiation model (BM-MSCs and C3H10T1/2). Accumulation of triglycerides by Oil Red O staining (Fig. 3f, g) and quantification of intracellular triglyceride content (Fig. 3h, i) explain that AMPK activator (metformin) delayed the differentiation to adipocytes even in the presence of adipogenic inducers, which is further clarified by western blot analysis showing the expression of other adipogenic marker, PPARγ (Fig. 3j, k). Real-time PCR analysis confirmed the expression of markers such as RUNX2, PPARγ and AdipoQ (Figure S2). To determine whether RUNX2 serine 118 residue was indeed phosphorylated by AMPK during differentiation, we differentiated normal MSCs (C3H10T1/2) with and without metformin, and immunoprecipitated the cell lysates with RUNX2 antibody. Results from the pulled immunocomplexes were probed with AMPK substrate antibody, suggesting that p-AMPK-mediated RUNX2-S118 phosphorylation is the key regulator of MSC differentiation process (Fig. 3l).

It is known that terminally differentiated osteocytes and myocytes can also dedifferentiate to adipocytes upon altered external stimuli, and that could be seen in the modified metabolic conditions^27^. In order to check the similar relationship of p-AMPK and RUNX2 even in such transdifferentiation process, we used transdifferentiation of U2OS (osteoblasts) to adipocytes, upon treatment with rosiglitazone showed a loss of AMPK activity and reduced RUNX2 level during differentiation process (Fig. 3m). Collectively, these findings suggest that AMPK-mediated RUNX2 phosphorylation is essential for maintaining the osteocytes, and loss of its activity could potentially lead to adipogenesis.

### High glucose-induced loss of RUNX2 phosphorylation is associated with loss of active AMPK

**Fig. 4:**
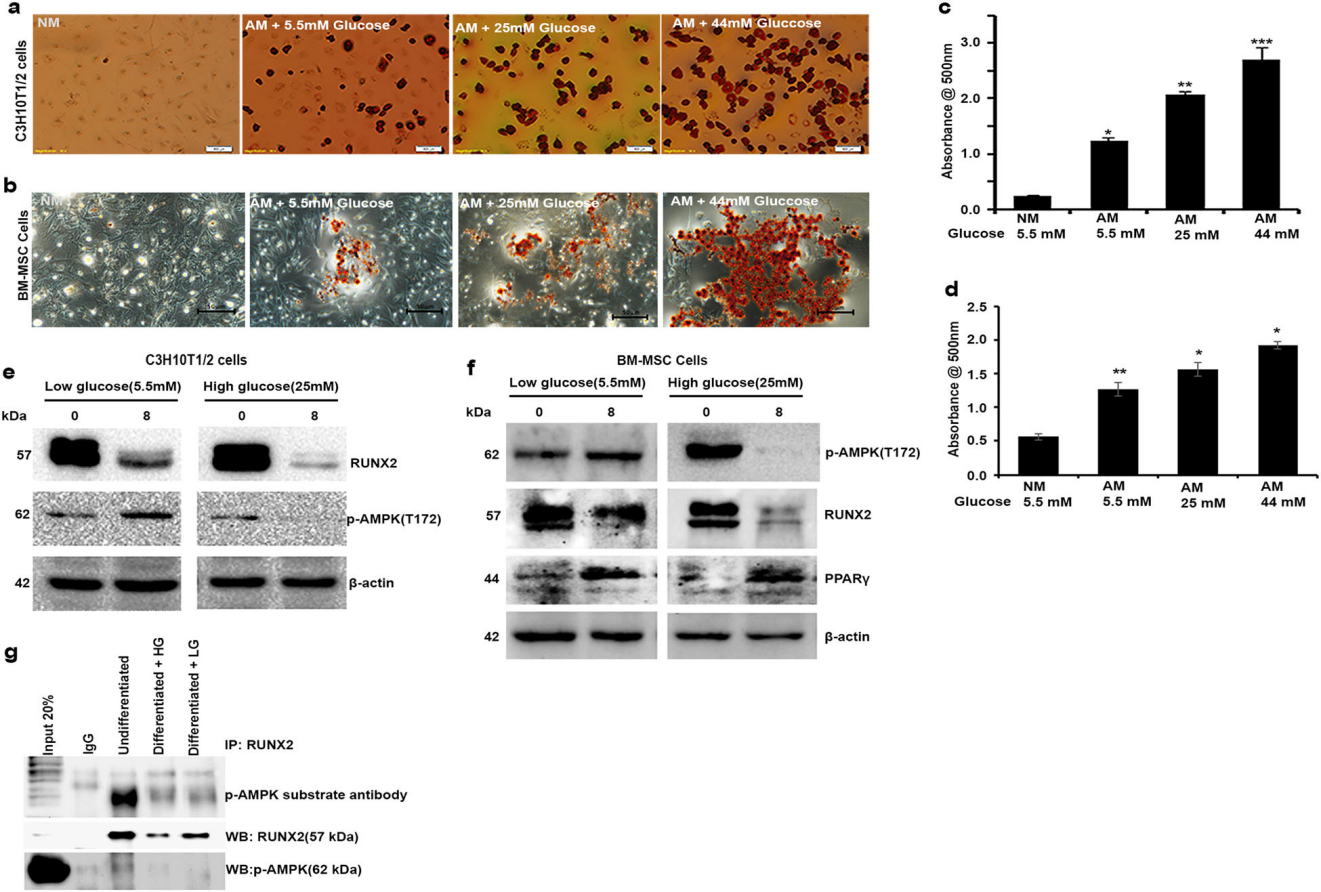
High glucose-induced adipogenic differentiation depends upon status of AMPK. MSCs (C3H10T1/2 & BM-MSCs) were exposed to various indicated concentrations of D (+) glucose along with adipogenic inducers. **a**, **b** Oil Red O stain shows glucose concentration (25 and 44 mM)-dependent increased adipogenesis compared with normal glucose (5 mM) and without adipogenic-induced medium (NM). **c**, **d** Representative picture showing the quantitative measurement of Oil Red O stain eluted with 100% isopropanol (*n* = 3). **e**, **f** Western blot analysis of p-AMPK and RUNX2 shows loss of AMPK activation and RUNX2 protein stability in high glucose-treated cells. **g** Immunoprecipitation analysis of undifferentiated and differentiated MSCs to adipogenesis with 5.5 mM (LG) and 25 mM (HG) concentrations of glucose showing the reduced levels of RUNX2, p-AMPK and RUNX2-S118 phosphorylation with p-AMPK substrate antibody. Mean ± S.E.M.; *n* = 3, *p < 0.1 versus NM+5.5 mM glucose control; **p < 0.05 versus NM+5.5 mM glucose control; ***p < 0.001 versus NM+5.5 mM glucose control

Reduced AMPK activity is associated with elevated levels of glucose in various cells and tissues^28^. As results from our earlier study suggest that AMPK activity is associated with RUNX2 protein level, here we have investigated the effects of high glucose (HG) on RUNX2-S118 phosphorylation and its effects on adipogenic model. MSCs (BM-MSCs and C3H10T1/2) were exposed to low glucose (5.5 mM), standard adipogenic-induced medium with 25 mM glucose and high glucose (44 mM) conditions as described in the materials and methods and observed for triglyceride formation and adipogenesis. Oil Red O staining (Fig. 4a–d) revealed that MSCs exposed to high glucose (25 and 44 mM) became more adipogenic with high levels of triglycerides as compared to low glucose with adipogenic induction medium (5.5 mM glucose). The p-AMPK and RUNX2 protein level were decreased in high glucose-induced (25 mM) differentiation when compared with normal level of glucose (5.5 mM) (Fig. 4e, f). The mRNA levels of RUNX2, PPARγ and AdipoQ were also correlated with adipogenesis (Figure S3), which shows chronic hyperglycemia favoring adipogenesis. Further immunoprecipitation with RUNX2 on lysates of MSCs (C3H10T1/2) differentiated with normal (5.5 mM) vs high (25 mM) glucose blots probed with p-AMPK substrate motif-specific antibody suggested that AMPK-mediated RUNX2-S118 phosphorylation is the key modulator of differentiation process during diabetes (Fig. 4g). Thus, these results suggest that exposure to physiological glucose levels maintains optimal AMPK activity and RUNX2 stabilization, which favors the homeostasis of osteogenesis vs adipogenesis.

### Streptozotocin-induced diabetic bone adipogenesis is abrogated by pharmacological activation of AMPK by metformin

**Fig. 5:**
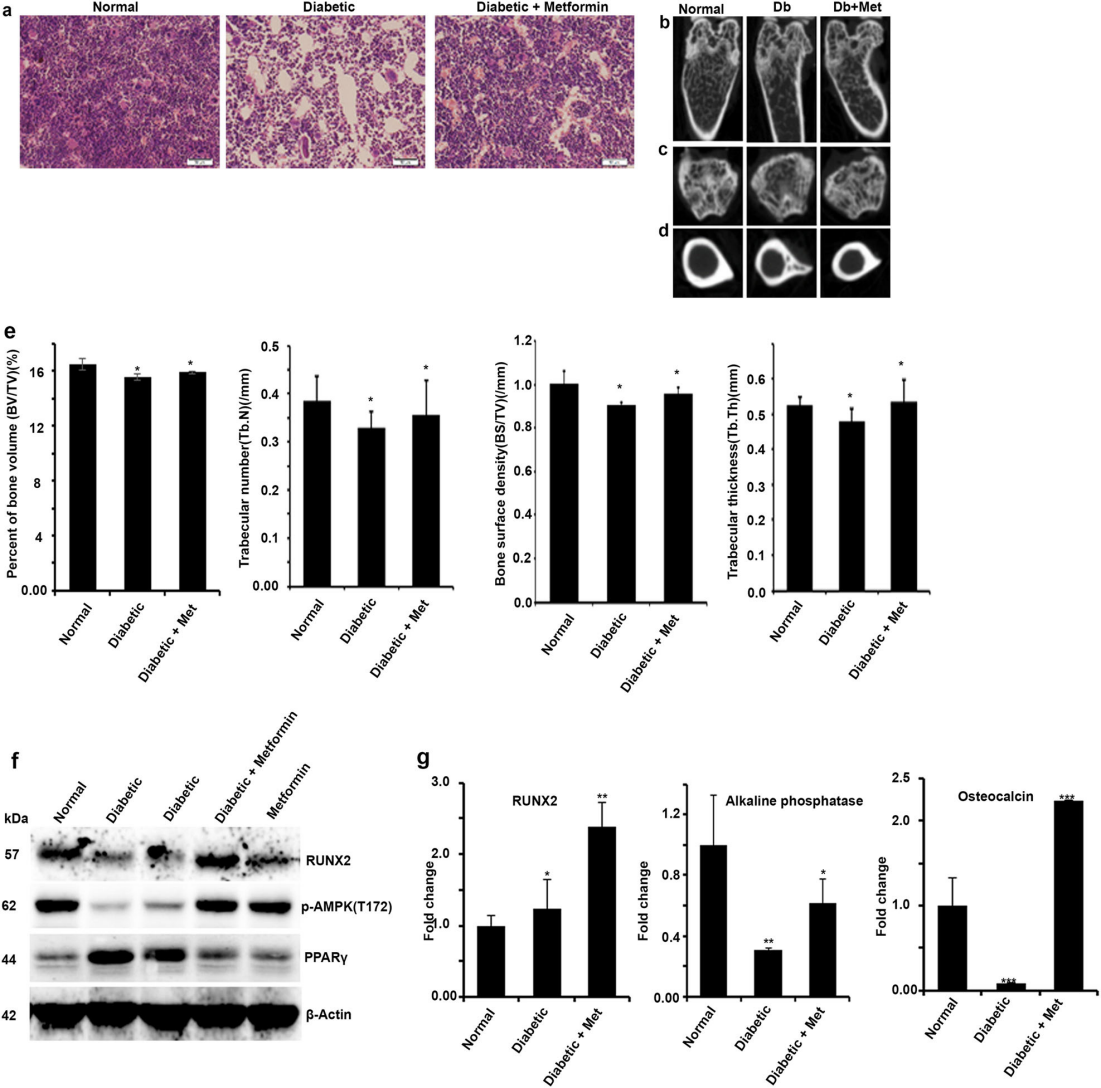
Streptozotocin-induced diabetic bone adipogenesis is abrogated by pharmacological activation of AMPK by metformin. Streptozotocin-induced diabetes mice were received with and without metformin for 10 weeks and bone adipogenesis was observed histologically on **a** H&E-stained cross-sections of tibia. The micro-CT image analysis showing **b** longitudinal, **c** tranaxial and **d** cortical midshaft on tibia of hind limbs. The histomorphometric parameters are expressed as **e** the change in percentage of bone volume (BV/TV), trabecular number (Tb.N), bone surface density (BS/TV) and trabecular thickness (Tb.Th). **f** Western blot analysis for p-AMPK, RUNX2 and PPARγ and **g** real-time PCR analysis for RUNX2, alkaline phosphatase and osteocalcin of bone marrow cell lysates. Mean ± S.E.M.; *n* = 3, *p < 0.1 versus Normal control; **p < 0.05 versus Normal control; ***p < 0.001 versus Normal control

Exposure to high glucose favors adipogenesis by modulating RUNX2 protein level by AMPK. In line with these observations, long-term treatment with AMPK activators metformin in chronic diabetic patients were shown to be less adipogenic and had healthy bones as compared to diabetic patients who were on other treatments^29^. Next, we explored the correlation between RUNX2 protein level and AMPK activity upon pharmacological exposure to metformin in streptozotocin-induced diabetic mice to reinstate their association in vivo. Histological sections of tibiae from streptozotocin-induced diabetic animals showed high levels of adipogenic as compared to control mice, whereas 10-week exposure of metformin abrogated diabetes-induced bone adipogenesis (Fig. 5a). To further explore these observations, micro-CT analysis of limb bones of mice treated with metformin for 10 weeks were visualized, which revealed that longitudinal and cross-sectional tibiae showed increased bone health in metformin-treated animals (Fig. 5b–e). Western blot and RT-PCR analysis of cells isolated from bone marrow showed that metformin treatment stabilized RUNX2 protein level and correlated with phospho-AMPK and other adipogenic vs osteogenic markers (Fig. 5f). The gene expression (mRNA) of osteocalcin and alkaline phosphatase levels in bone marrow cells explained the decreased osteogenic markers in diabetes except RUNX2 (Fig. 5g). Collectively, these findings for the first time conclude that correlation of healthy bones in patients who are on metformin is through stabilization of RUNX2 via active AMPK and loss of this balance leads to bone adipogenesis.

### RUNX2 ubiquitination is linked to RUNX2- S118 phosphorylation

**Fig. 6:**
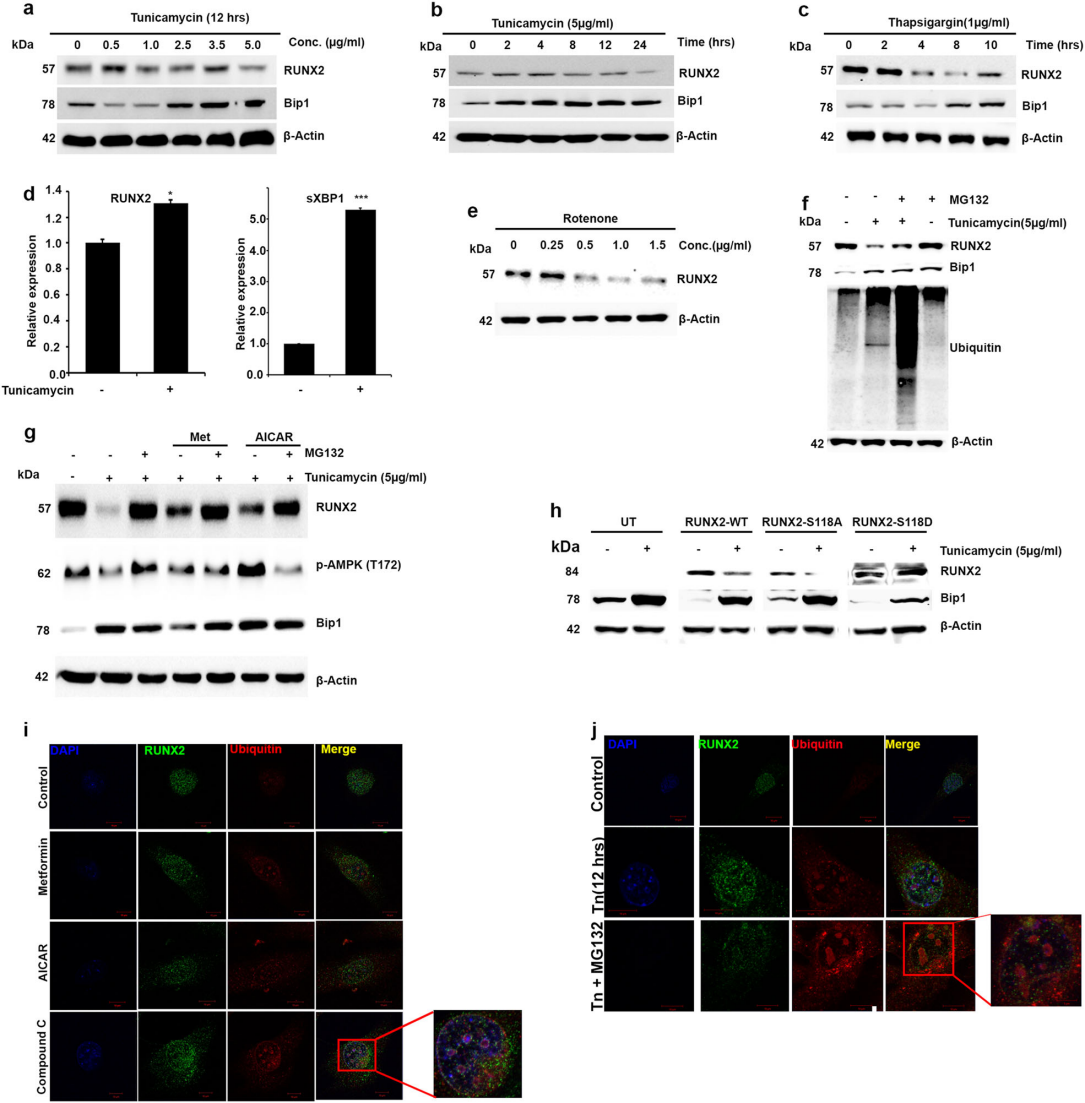
RUNX2 ubiquitination is linked to RUNX2-S118 phosphorylation. Western blot analysis of murine MSCs (C3H10T1/2) showing RUNX2, Bip1 protein levels after treatment with different **a** concentrations, **b** time duration of tunicamycin and **c** thapsigargin. **d** Real-time PCR analysis of RUNX2 showing increased RNA levels with tunicamycin similar to stress response genes sXBP1. **e** Treatment with rotenone (oxidative stress inducer) also shows similar expression of RUNX2. **f** Proteosomal inhibitor MG132 abrogated tunicamycin-induced loss of RUNX2 expression and association of ubiquitination. **g** Pre-activation of AMPK or treatment with MG132 (for 1 h) followed by tunicamycin (5 µg/ml) treatment for 12 h showing AMPK activation inhibits the effects of tunicamycin similar to MG132. **h** Western blot analysis showing transiently transfected phosphomimetic mutant RUNX2-S118D is resistant to stress-induced ubiquitination in HEK 293T cells. Immunofluorescence analysis showing the effects of colocalization of RUNX2 with ubiquitin **i** in the presence of AMPK activators (metformin and AICAR) and compound C and **j** tunicamycin with and without MG132. Mean ± S.E.M.; *n* = 3, *p < 0.1 versus untreated control; ***p < 0.001 versus untreated control

Exposure to high glucose and streptozotocin-induced diabetes models shows that stabilization of RUNX2 depends upon AMPK activation, and our in vitro kinase assays showed that AMPK phosphorylates RUNX2 at S118. However, the role of serine 118 on the synergistic correlation of AMPK and stabilization of RUNX2 is still uncertain. It is well established that impaired glucose tolerance and type 2 diabetes mellitus induces endoplasmic reticulum (ER)^30^ and oxidative stress^31^. Here, we examined the effects of ER and oxidative stress on RUNX2 protein level and the mechanisms involved in RUNX2 stabilization. We first studied the effect of tunicamycin, an ER stress inducer on MSCs (C3H10T1/2) and observed that RUNX2 protein levels are decreased in a time- and dose-dependent manner. However, RNA levels of RUNX2 were unchanged with tunicamycin treatment (Fig. 6a–d). Similarly, another correlation was observed when they were treated with oxidative stress inducers (rotenone) (Fig. 6e). However, treatment with proteosomal inhibitor (MG132) resulted in inhibition of stress-induced reduction in the RUNX2 protein level, indicating that RUNX2 may be subjected to proteosomal degradation under physiological stress conditions (Fig. 6f). To test whether ubiquitin-mediated RUNX2 degradation is in agreement with active AMPK, cells were treated with AMPK activators along with and without proteosomal inhibitors and subjected to western blot analysis. Treatment with AMPK activators maintained the level of RUNX2 similar to treatment with MG132 alone (Fig. 6g). Collectively, it is revealed that active AMPK is critical for stabilizing RUNX2 protein level from proteosomal degradation, which could be due to phosphorylation of S118RUNX2. Similar results were pronounced on myoblast cells (C2C12) (Figure S4 a-g). To confirm this evidential hypothesis, we treated HEK 293T cells overexpressing RUNX2-WT, RUNX2-S118A and RUNX2-S118D with and without tunicamycin. It was revealed that ER stress-induced degradation of RUNX2 is not affected by phosphomimetic RUNX2-S118D-overexpressed cells, whereas a degradation was observed in RUNX2-WT and RUNX2-S118A (Fig. 6h)-expressed cells. Further immunofluorescence studies showed colocalization of ubiquitin with RUNX2 in the presence of an AMPK inhibitor (compound C) or tunicamycin, but not in metformin-treated MSCs (Fig. 6i, j) and C2C12 cells (Figure S4 h-k).

## Discussion

Management of diabetes and obesity have become challenging in the field of metabolic disorders, due to widespread occurrence of diabetes around the world, especially in India and China^32,33^. Bone formation and resorption is a tightly regulated process, which is important for continuous remodeling and homeostasis^34^. Years of research has made it clear that the osteogenic and adipogenic processes from MSCs are reciprocally regulated;^1^ for instance, BMP2 at high concentration induces osteogenesis whereas in low concentrations it induces adipogenesis^35^. AMPK is sufficient to stimulate osteogenesis of MC3T3-E1 cells at the cost of adipogenesis, but lack of AMPK leads to adipogenesis^15^. However, the exact molecular mechanisms underlying the reciprocal relationship in cell fate commitment and transdifferentiation during altered metabolic (pathogenic) conditions are yet to be elucidated.

Our findings mechanistically demonstrate that RUNX2 phosphorylation is critical in osteogenic commitment; however, loss of RUNX2 may activate the adipogenic process. Our results also establish for the first time that RUNX2 is a metabolically regulated substrate for AMPK and is shown to be phosphorylated at RUNX2-S118. To the best of our knowledge, this is the first report showing direct relation of AMPK-mediated RUNX2 phosphorylation at serine 118. To examine the functional relationship between RUNX2 phosphorylation and differentiation of MSCs, distinctive models were used. MSCs (BM-MSCs and C3H10T1/2) to osteocytes vs adipocytes shows that activation of AMPK (T172) is correlated with stabilization of RUNX2 and its S118 phosphorylation as shown in immunoprecipitation studies (Fig. 3h). Whereas, loss of p-AMPK is followed by decreased RUNX2 protein level in adipogenesis, but without affecting RUNX2 mRNA level (Fig. 3d, e). Similar correlation was evident in transdifferentiation models, viz., myocytes to adipocytes, osteocytes to adipocytes and pre-adipocytes to adipocytes. These studies correlate with the well-thought hypothesis that active AMPK helps in reducing adipogenesis and maintaining healthy bone, and also explains that RUNX2 being a substrate of AMPK may act as a regulatory connection between energy homeostasis and osteoblast development. The transdifferentiation studies confirm the adipogenic process in altered metabolic conditions; however, it should also be carefully considered that metformin and compound C studies have revealed that AMPK may maintain a homeostasis of osteocytes vs adipocytes where low level of AMPK may favor adipogenesis, whereas high level of AMPK may lead to osteocytes by stabilizing RUNX2 protein. This was evident from the fact that there was minimal activity of p-AMPK (T172) all the time as seen from our studies as well as by other investigators^13^. As mRNA levels of RUNX2 are maintained in both the differentiation processes, there is an indication that dynamic and sequential regulation at translational or post-translational level may exist.

RUNX2 and PPARγ are the two critical transcription regulators of mesenchymal differentiation of osteogenesis and adipogenesis respectively^36,37^. RUNX2^−/−^ mice failed to differentiate into osteocytes but spontaneously committed towards adipogenesis ^36^, whereas PPARγ^−/−^ mice showed impaired adipogenesis but formed osteocytes^10^. Differentiation models explained that PPARγ is expressed early in the adipocyte differentiation program. Similarly, RUNX2 expression is seen all through mesenchymal condensation representing the common precursors of osteoblasts and chondrocytes. RUNX2 is mainly required for the commitment of the mesenchymal cells towards the osteogenic lineage that is for formation of immature osteoblast, but may not be critical for further maturation of osteoblasts to osteoclasts. In fact, RUNX2 inhibits maturation process^38^. These demonstrations indicate that RUNX2 is indispensable for bone formation. The recent studies demonstrated that glucose but not RUNX2 is indispensable for bone formation. However, this discrepancy is mainly due to variations in cellular contexts, because glucose seems to promote maturation of bone by promoting type I collagen synthesis^39^ whereas RUNX2 acts at upstream to maturation^38^. This shows that specific regulatory signals are involved in the differential commitment process.

However, the RUNX2 expression is maintained in cells derived from osteoblastogenesis but lost in chondrocyte-derived cells. Thus, RUNX2 may not be critical for later stage maturation of osteoblasts to osteoclasts. These demonstrations indicate that regulatory signals are involved in the differential commitment process. It is known that increased PPARγ activity is observed during decreased level of AMPK expression and favors fat cell development. Our results further showed that active AMPK is responsible for stability of RUNX2 by regulating it at post-translational level, and these observations are correlated with commitment of osteogenesis. Thus, these results give us a hint that AMPK senses the cellular metabolic energy and regulates the homeostasis of RUNX2 vs PPARγ, which directs the MSCs into osteoblasts vs adipogenic commitment.

From a clinical perspective, hyperglycemia positively regulates adipogenic differentiation by inhibiting osteogenic variation in muscle-derived stem cells^40^ and is also shown to activate polyol pathway by decreasing the DNA-binding affinity of RUNX2^41^. Metformin-induced osteogenesis and mineralization in OCT-1-expressing induced pluripotent stem cell-derived MSCs demonstrated its potential use for enhanced bone and periodontal regeneration in diabetic patients^42^. Epidemiological clinical data suggest that long-term administration of metformin reduces bone adipogenesis and resorption^29^ similarly, use of thiazolidinediones has been reported to show reduced bone mineral density and activation of PPARγ-induced osteoclastogenesis^43^. Based on these clinical and experimental evidences, we investigated for an in vivo role of AMPK and bone adipogenesis in connection with RUNX2 phosphorylation using streptozotocin-induced diabetic mice model.

The micro-CT image analysis of hind limbs shows comparatively better healthy bone architecture in the metformin group compared to the diabetic controls (Fig. 5). This analysis is correlated with the active AMPK and RUNX2 protein levels in bone marrow cells and further correlated with bone adipogenesis in sections of tibia. Our studies are in line with earlier observations that in vitro model of bone formation using primary osteoblasts shows that metformin and AICAR treatments stimulated bone nodule formation, whereas compound C ameliorated the mineralization^44^. It has been demonstrated that RUNX2 expression is controlled by both transcriptional and translational levels. Our real-time PCR experiments highlight the maintenance of RUNX2 mRNA level in both in vitro adipogenic differentiation and diabetes-induced streptozotocin model of bone marrow cells. Significantly low level of RUNX2 protein gives us an indication of the existence of regulation at translational level during this differentiation process. It has been shown that ubiquitin–proteasome-mediated RUNX2 degradation is regulated by E3 ligases, namely Smurf1^18^, WWP1^45^, CHIP^46^, Fbw7 and Skp2^20^. Studies have also demonstrated that these ligases negatively regulate RUNX2 activity and the osteogenic process. Activation of AMPK by either metformin or AICAR inhibits the tunicamycin -induced RUNX2 degradation. Brief transfection studies using WT, S118A and S118D variants of RUNX2 in HEK cells demonstrated that S118 phosphorylation is critical for ubiquitination process in various experimental set-ups. It has been shown that amino acid region 97–341 is important for Skp2, Fbw7 and WWP1 physical interaction and ubiquitination process. Collectively, we may conclude that S118 phosphorylation in AMPK substrate binding domain (113–118) is important for ubiquitin-mediated regulation. RUNX2 is also known to be regulated by GSK3β^47,48^, JNK^49^, Cdk4^50^, Cdk1^51^ and cdc2^41^. Although glycogen synthase kinase-3β (GSK3β) and c-Jun N-terminal kinase (JNK) share the same serine 118 as the kinase target for phosphorylation, its functional consequences in relation with differentiation were not established. However, genetic knockdown of GSK3β has shown that RUNX2 phosphorylation by GSK3β inhibits the transcriptional activity and osteogenesis. These studies have shown that specific phosphorylation occurs in inhibitory transactivation domain rather than DNA-binding domain where S118 is sited. Similarly, studies have shown that metformin suppresses adipogenesis by both AMPK-dependent and -independent mechanisms, whereas in exercise-induced model, mTORC2 has shown to be critical in muscle glucose uptake independent of AMPK^13^. These findings demonstrate that interpretation of metformin actions on osteogenesis vs adipogenesis needs to be taken into consideration; however, AMPK has proven critical in maintaining the homeostasis.

**Fig. 7:**
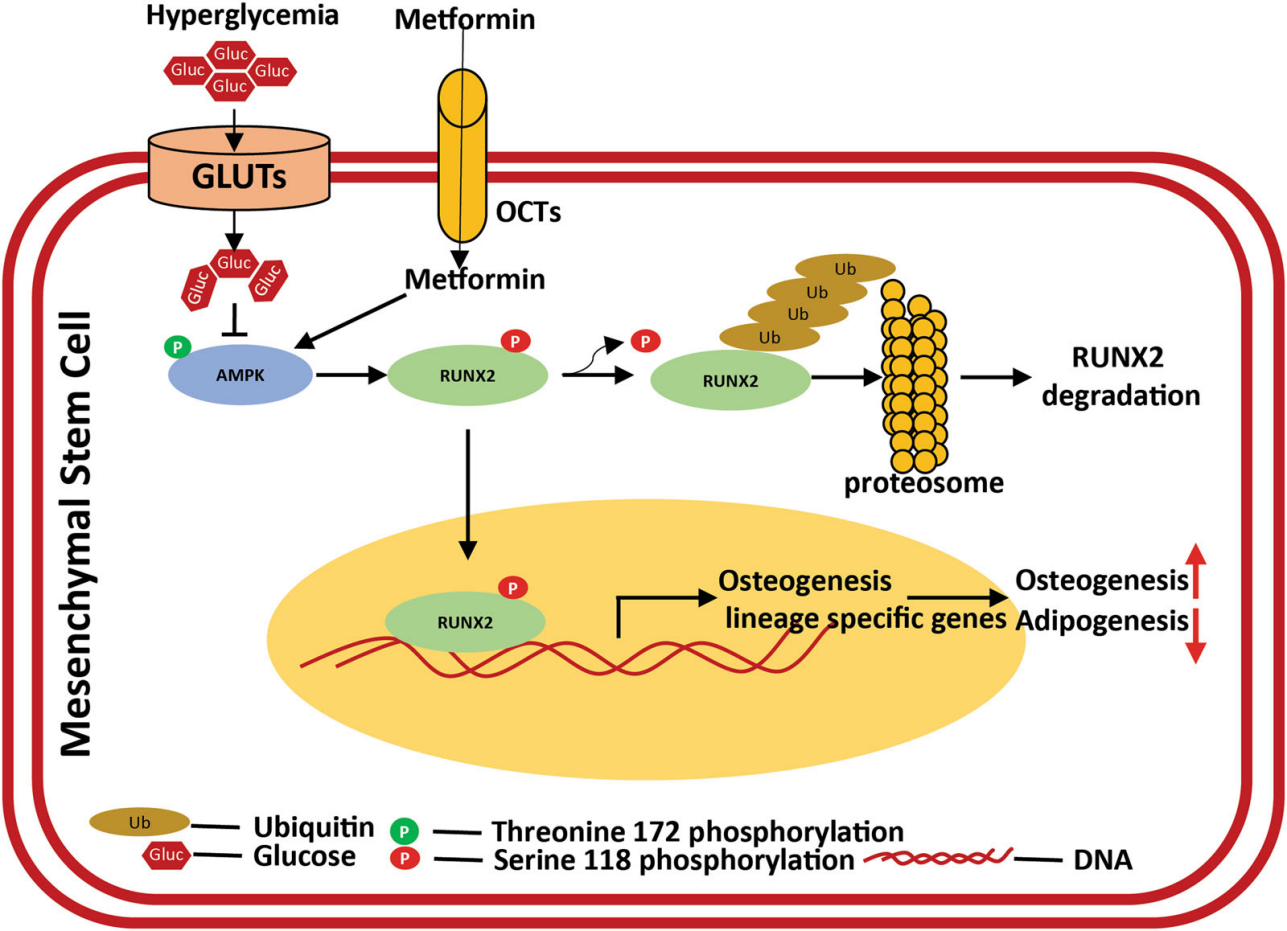
Schematic representation of AMPK-mediated RUNX2 regulation and its mechanism in differentiation. The schematic mechanism representing the molecular insights of multipotent MSCs differentiation of osteogenesis vs adipogenesis. AMPK is a physiological sensor and regulates energy homeostasis through multiple intermediates. Under altered metabolic conditions, such as hyperglycemia, with reduced active AMPK favors adipogenesis. The homeostasis in differentiation of osteogenesis vs adipogenesis is due to regulation of RUNX2, which is known to be a master regulator of osteogenesis. This regulation is maintained by phosphorylation of RUNX2-S118, by stabilizing its protein levels from proteosomal degradation (ubiquitination). This could be a plausible phenomenon, where AMPK activators such as metformin etc. are shown to have healthy bones, especially under metabolic stress conditions such as diabetes

In conclusion, our results demonstrated that RUNX2 is a novel substrate of AMPK and RUNX2-S118 phosphorylation, which also protects it from ubiquitination. Stress (tunicamycin, hyperglycemia)-induced degradation of RUNX2 depends upon active level of AMPK. Clinical correlation of metformin restricts diabetes-induced bone adipogenesis, might be due to stabilization of RUNX2 (Fig. 7). Overall, AMPK plays a critical role in maintaining the RUNX2 and PPARγ expression and cellular energy levels, thereby regulating the differentiation of osteogenesis vs adipogenesis.

## Electronic supplementary material

Table S1

Figure S1, S2, S3

Figure S4

Supplementary figure legends
