# Supplementary material for: A novel phosphorylation by AMP-activated kinase regulates RUNX2 from ubiquitination in osteogenesis over adipogenesis
Source: Cell Death Dis. 2018 Jul 9;9(7):754. doi: 10.1038/s41419-018-0791-7 (PMC6037667; doi:10.1038/s41419-018-0791-7)
Supplement: Supplementary file 1 — Table S1 [file 41419_2018_791_MOESM1_ESM.docx]

| **S.No** | **Primer Name** | **Primer Sequence** |
| --- | --- | --- |
| 1 | RUNX2-WT-Fwd | 5’-CCGCTCGAGACCATGGCATCAAACAGCCTCTTCAGC-3’ |
| 2 | RUNX2-WT-Rev | 5’-GGAAGATCTCGATATGGTCGCCAAACAGATTCATC-3’ |
| 3 | RUNX2-S118A-Fwd | 5’-GTCCGCACCGACGCCCCCAACTTCCTG-3’ |
| 4 | RUNX2- S118A -Rev | 5’-CAGGAAGTTGGGGGCGTCGGTGCGGAC-3’ |
| 5 | RUNX2-Fwd-RT | 5’-CCGGTCTCCTTCCAGGAT-3’ |
| 6 | RUNX2-Rev-RT | 5’-GGGAACTGCTGTGGCTTC-3’ |
| 7 | Osteocalcin-Fwd-RT | 5’-CCAAGCAGGAGGGCAATA-3’ |
| 8 | Osteocalcin-Rev-RT | 5’-TCGTCACAAGCAGGGTCA-3’ |
| 9 | ALP-Fwd-RT | 5’-CCCCATGTGATGGCGTAT-3’ |
| 10 | ALP-Rev-RT | 5’-CGGTAGGGAGAGCACAGC-3’ |
| 11 | PPARγ-Fwd-RT | 5’-GGAAGACCACTCGCATTCCTT-3’ |
| 12 | PPARγ-Rev-RT | 5’-TCGCACTTTGGTATTCTTGGAG-3’ |
| 13 | AdipoQ-Fwd-RT | 5’-TGTTCCTCTTAATCCTGCCCA-3’ |
| 14 | AdipoQ-Rev-RT | 5’-CCAACCTGCACAAGTTCCCTT-3’ |
| 15 | sXBP1-Fwd-RT | 5’-TGAGAACCAGGAGTTAAGAACACGC-3’ |
| 16 | sXBP1-Rev-RT | 5’-CCTGCACCTGCTGCGGAC-3’ |
| 17 | Actin-Fwd-RT | 5’-AGTACCCCATTGAACGC-3’ |
| 18  19  20 | Actin-Rev-RT  C/EBP beta-Fwd- RT  C/EBP beta-Rev-RT | 5’-TGTCAGCAATGCCTGGGTAC-3’  5'-GGGTTGTTGATGTTTTTGGTTT -3'  5'-GAAACGGAAAAGGTTCTCAAAA -3' |

**Table S1: Primers list**
