# Supplementary figures and images for: A novel phosphorylation by AMP-activated kinase regulates RUNX2 from ubiquitination in osteogenesis over adipogenesis
Source: Cell Death Dis. 2018 Jul 9;9(7):754. doi: 10.1038/s41419-018-0791-7 (PMC6037667; doi:10.1038/s41419-018-0791-7)

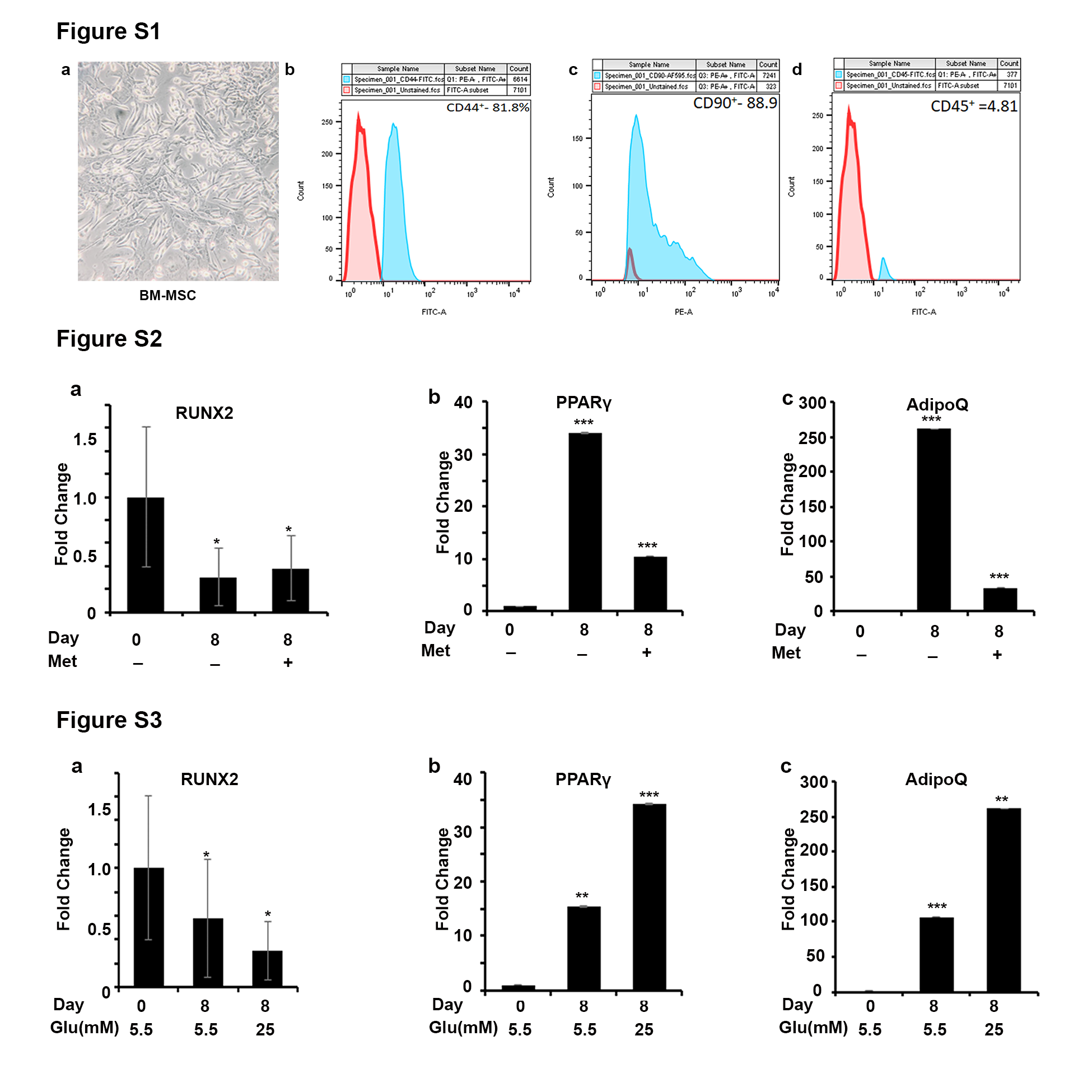

Supplement: Supplementary file 2 — Figure S1, S2, S3 [file 41419_2018_791_MOESM2_ESM.tif]

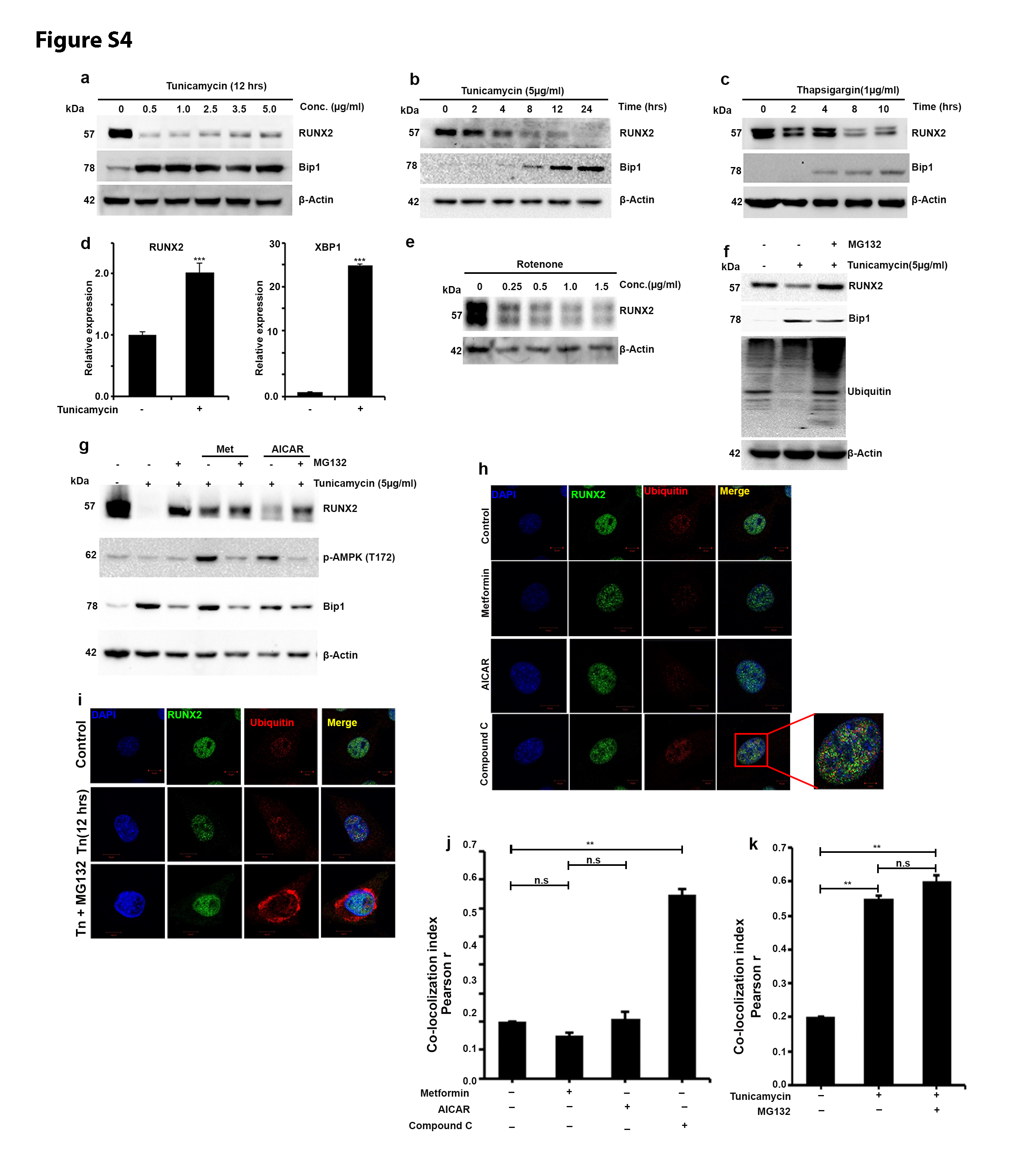

Supplement: Supplementary file 3 — Figure S4 [file 41419_2018_791_MOESM3_ESM.tif]
