# Supplementary material for: A novel phosphorylation by AMP-activated kinase regulates RUNX2 from ubiquitination in osteogenesis over adipogenesis
Source: Cell Death Dis. 2018 Jul 9;9(7):754. doi: 10.1038/s41419-018-0791-7 (PMC6037667; doi:10.1038/s41419-018-0791-7)
Supplement: Supplementary file 4 — Supplementary figure legends [file 41419_2018_791_MOESM4_ESM.docx]

**Supplementary Figure Legends**

**Figure S1**: Characterization of BM-MSCs by using surface antigens: a) morphological characteristics of BM-MSCs at passage 3. Flow cytometry analysis showed 81.8% positive for (b) CD44 (c) 88.9% for CD90 and (d) 4.81% for CD45.

**Figure S2:** The Real-time PCR analysis of RUNX2 (Figure S2a), PPARγ (Figure S2b) and AdipoQ (Adiponectin) (Figure S2c) gene expression in C3H10T1/2. The transcript levels were quantified from cDNA of fully differentiated vs undifferentiated using with and without metformin (5 mM) treatments. Relative gene expression levels were normalized to the corresponding β-actin gene expression. The fold change was calculated by log ratios, and error bars represents the standard deviation of three independent experiments performed in triplicate. Mean ± S.E.M.; n=3, *p<0.1 versus Undifferentiated control, ***p<0.001 versus Undifferentiated control.

**Figure S3:** The Real-time PCR analysis of RUNX2 (Figure S3a), PPARγ (Figure S3b) and AdipoQ (Adiponectin) (Figure S3c) gene expression in C3H10T1/2. The transcript levels were quantified from cDNA of fully differentiated vs undifferentiated groups with variable concentrations of normal (5.5 mM) and high glucose (25 mM). Relative gene expression levels were normalized to the corresponding β-actin gene expression. The fold change was calculated by log ratios, and error bars represents the standard deviation of three independent experiments performed in triplicate. Mean ± S.E.M.; n=3, *p<0.1 versus Undifferentiated control; **p<0.01 versus Undifferentiated control; ***p<0.001 versus Undifferentiated control.

**Figure S4: RUNX2 ubiquitination is linked to RUNX2-S118 phosphorylation:**

Western blot analysis of myoblast cells (C2C12) showing RUNX2, Bip1 protein levels after treatment with different **(a)** concentrations, **(b)** time duration of tunicamycin and (**c)** thapsigargin. **(d)** Real-time PCR analysis of RUNX2 showing increased RNA levels with tunicamycin similar to stress response genes sXBP1. **(e)** Treatment with rotenone (oxidative stress inducer) also shows similar expression of RUNX2. **(f)** Proteosomal inhibitor MG-132, abrogated tunicamycin-induced loss of RUNX2 expression and association of ubiquitination **(g)** Pre-activation of AMPK or treatment with MG132 (for 1 hr) followed by tunicamycin (5µg/ml) treatment for 12 hr showing AMPK activation inhibits the effects of tunicamycin similar to MG132. Immunofluorescence analysis showing the effects of colocalization of RUNX2 with ubiquitin **(h)** in presence of AMPK activators (Metformin and AICAR) and Compound C and **(i)** tunicamycin with and without MG132. Quantification of immunofluorescence analysis images (3 fields) showing colocalization of RUNX2 with ubiquitin **(j)** Pre-activation of AMPK or treatment with MG132, for 1 hr and induction of stress by tunicamycin (5µg/ml) for 12 hr showing that AMPK activation inhibits the **(k)** effects of tunicamycin similar to MG132. The data represented as Mean ± S.E.M.; n=3, the P value less than 0.05 is considered as significant **p<0.01, n.s = not significant.
